# Sarcoidosis Presenting as Longitudinally Extensive Transverse Myelitis: A Case Report

**DOI:** 10.5334/jbsr.3479

**Published:** 2024-06-12

**Authors:** Joudia Touri, Abdellatif Bali, Jean-François Poma

**Affiliations:** 1 Department of Neurosurgery, Cliniques Universitaires Saint-Luc, Université Catholique de Louvain, Brussels, Belgium; 2 Department of Radiology, Clinique Saint-Jean, Brussels, Belgium; 3 Department of Neurology, Clinique Saint-Jean, Brussels, Belgium

**Keywords:** sarcoidosis, neurosarcoidosis, transverse myelitis

## Abstract

Spinal cord sarcoidosis is a rare condition that can present as a longitudinally extensive transverse myelitis. Current imaging may suggest this pathology, but the final diagnosis relies on the histologic findings.

*Teaching point:* Considering neurosarcoidosis in the differential diagnosis of longitudinally extensive transverse myelitis.

## Introduction

Sarcoidosis is a multisystemic disorder frequently affecting the lungs, mediastinal lymph nodes, uvea, and skin. In up to 15% of cases, the nervous system can be involved [1]. Intracranial manifestations of neurosarcoidosis (NS) are most common, whereas spinal cord sarcoidosis (SCS) represents 16% to 28% [1–3] and occurs in less than 1% of patients with systemic disease [3].

We report a case of longitudinally extensive transverse myelitis as the initial manifestation of sarcoidosis, presenting exclusively with subacute sensory deficits.

## Case Report

A 47-year-old man, without notable medical history, was admitted with a 2-month history of progressive and symmetrical paresthesia involving both hands and intermittent neck pain. The neurological examination was normal.

Routine labs and electroencephalogram were unremarkable. Cerebrospinal fluid (CSF) analysis demonstrated elevated protein levels (73 mg/dL) and specific oligoclonal bands; glucose and IgG index were normal.

**Figure 1:**
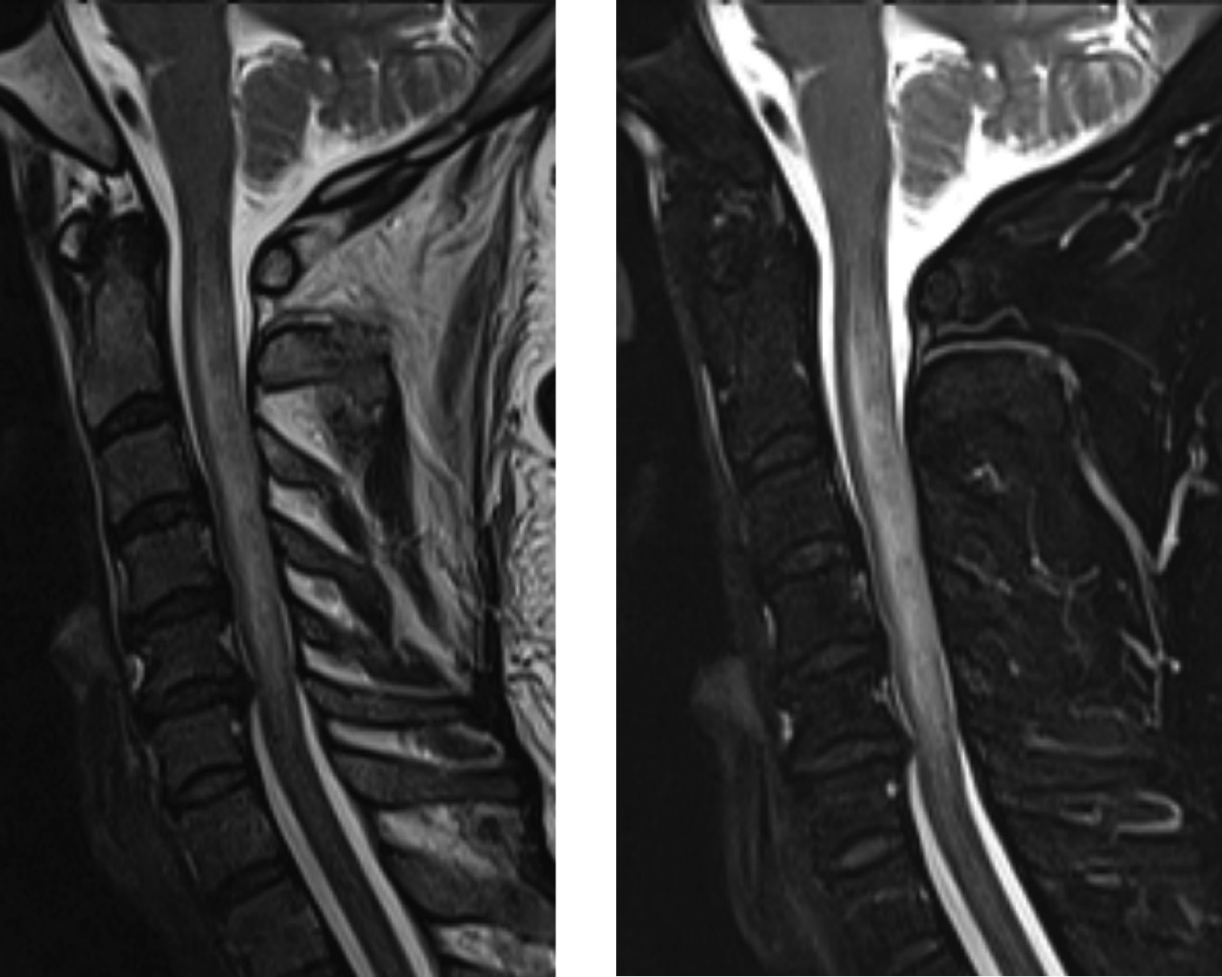
Sagittal TSE T2 (left) and T2 Dixon fat-saturated (right) MRI sequences of the cervical spinal show a swollen spinal cord associated with a T2-hyperintense signal extending from C2 to C5.

**Figure 2:**
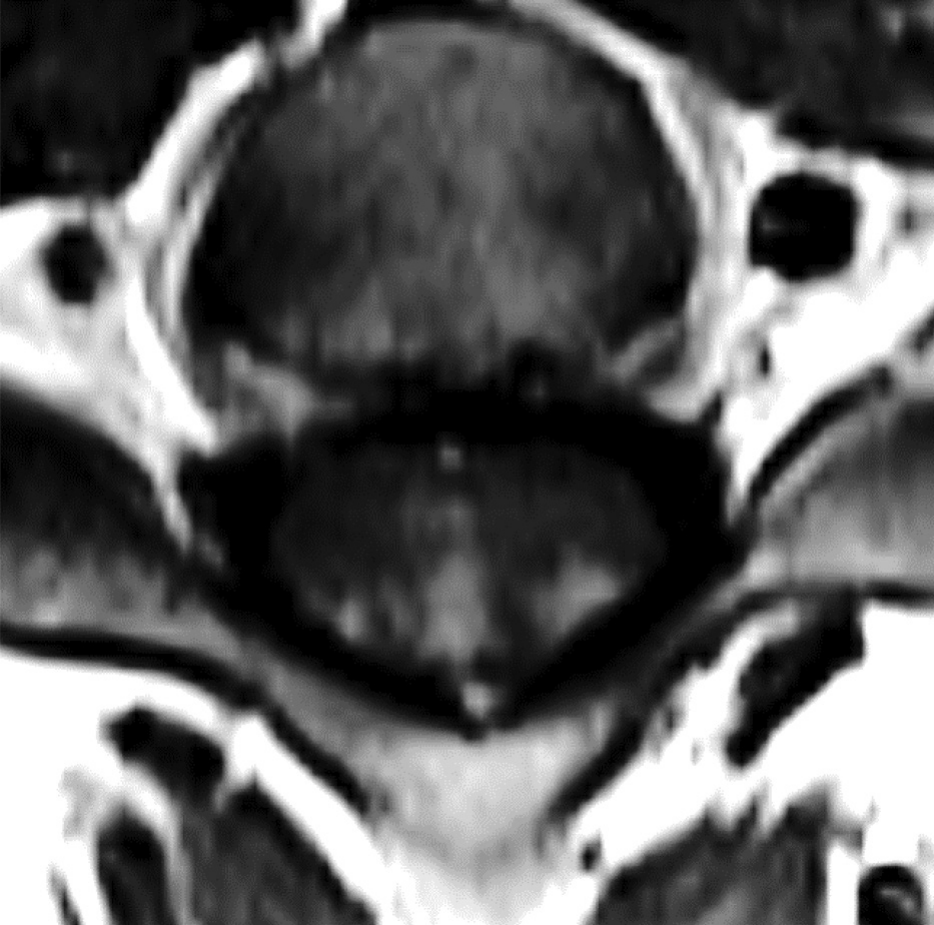
Axial TSE T1 sequence after administration of intravenous gadolinium shows a fork-like ‘trident’ contrast enhancement of the dorsal side of the spinal cord.

Brain magnetic resonance imaging (MRI) was normal. MRI of the spine demonstrated a T2-hyperintense elongated central cord lesion extending from C2 to C6 (Figure 1), with the typical “trident sign” suggestive of NS (Figure 2).

Total body FDG PET-CT showed hypermetabolic mediastinal lymphadenopathies and a moderately increased metabolism in the intramedullary cervical region from C3 to C5.

A mediastinal lymph node biopsy confirmed noncaseating granulomas without evidence of malignancy or infection, which led to the final diagnosis of NS. No medullary biopsy was undertaken as the risk was deemed higher than the benefit. The patient received intravenous methylprednisolone 1 g daily for 3 days, followed by oral maintenance corticosteroid therapy, which was subsequently switched to mycophenolic acid.

At 1-month follow-up, the sensory complaints had improved. Medullar MRI showed a significant decrease in the T2 hyperintense lesion, as well as a fading of the enhancement. It remained stable for 3 months. At 4 months, FDG PET-CT showed no metabolic active lesions.

## Discussion

The scarcity of NS and the wide range of its presentations can hinder its diagnosis, and the absence of any conclusive test makes it even more challenging. Clinical, imaging, and histologic evidence are key to establish the diagnosis.

SCS affects less than 1% of patients with systemic disease [3]. SCS presents as an intramedullary lesion in 35% of cases. Longitudinally extensive transverse myelitis is defined as spinal cord inflammation that extends longitudinally over at least three vertebral segments. Intramedullary NS presents as such in 77% of cases. There is an affinity for cervical and thoracic levels, and lesions often extend to several spinal segments [2, 4].

Intramedullary NS may be the initial manifestation of sarcoidosis. These patients present with insidious and progressive sensory symptoms [2–4].

MRI is the gold standard imaging technique for the diagnosis of SCS. In the early stages, SCS can present as a linear leptomeningeal contrast enhancement. It subsequently evolves into a centripetal spread of the inflammatory process along the Virchow–Robin spaces to manifest as parenchymal swelling and cord enhancement, corresponding to transverse myelitis, hyperintense on T2-weighted images, often stretching over multiple spinal segments [5]. In the third phase, the swelling decreases while focal enhancing regions may persist. The final stage is characterized by a resolution of the inflammatory process without sequelae or leaving behind an atrophic spinal cord [3].

CSF analysis is abnormal in approximately 80% of patients with SCS [5]. The findings most consistently demonstrate lymphocytic pleocytosis, elevated proteins, and hypoglycorrhachia. It might reveal an increased immunoglobulin G index and oligoclonal bands [1, 2, 4, 5]. These findings are nonspecific, but CSF analysis remains important to exclude infectious and neoplastic processes.

The diagnosis of sarcoidosis relies on the noncaseating granulomas found on the biopsies. Given the risks of procedural morbidity of spinal cord biopsy, we often fall back on histologic evidence from accessible extraneural lesions [1]. Computed tomography and FDG PET-CT help identify active systemic disease, usually clinically silent [2], and target confirmatory biopsy sites [3], the lungs and intrathoracic lymph nodes being the most common ones. In cases where clinical decline is observed despite treatment, a neural tissue biopsy may be necessary.

SCS carries a poorer prognosis than other manifestations of NS [5]. Prompt recognition and treatment can improve the course of the disease. Corticosteroids remain the first-line therapy [1, 4]. SCS can be steroid-refractory and might require additional immunosuppressive therapy. Surgical excision, partial or extensive, is not recommended as it may cause postoperative deterioration in neurological function.

Our patient improved clinically and radiologically, but MRI findings in response to corticosteroids do not always match the clinical response [2, 3, 5].

## Conclusion

Management of SCS has been experience-based rather than evidence-based due to the scarcity of the condition. NS should be considered in the differential diagnosis of longitudinally extensive transverse myelitis, even in the absence of previously diagnosed sarcoidosis. Current imaging may suggest this pathology, but the diagnosis relies mostly on the histologic findings of an extraneural biopsy. Corticosteroids remain the cornerstone of therapy.
